# PhyloPlus: a Universal Tool for Phylogenetic Interrogation of Metagenomic Communities

**DOI:** 10.1128/mbio.03455-22

**Published:** 2023-01-16

**Authors:** Xinyang Huang, David L. Erickson, Jianghong Meng

**Affiliations:** a Joint Institute for Food Safety and Applied Nutrition, Center for Food Safety Security Systems, University of Maryland, College Park, Maryland, USA; b Department of Nutrition and Food Science, University of Maryland, College Park, Maryland, USA; University of California, Irvine

**Keywords:** diversity, metagenomics, microbial genomics, microbiome, phylogeny

## Abstract

Phylogeny is a powerful tool that can be incorporated into quantitative descriptions of community diversity, yet its use has been limited largely due to the difficulty in constructing phylogenies which incorporate the wide genomic diversity of microbial communities. Here, we describe the development of a web portal, PhyloPlus, which enables users to generate customized phylogenies that may be applied to any bacterial or archaeal communities. We demonstrate the power of phylogeny by comparing metrics that employ phylogeny with those that do not when applied to data sets from two metagenomic studies (fermented food, *n* = 58; human microbiome, *n* = 60). This example shows how inclusion of all bacterial species identified by taxonomic classifiers (Kraken2 and Kaiju) made the phylogeny perfectly congruent to the corresponding classification outputs. Our phylogeny-based approach also enabled the construction of more constrained null models which (i) shed light into community structure and (ii) minimize potential inflation of type I errors. Construction of such null models allowed for the observation of under-dispersion in 44 (75.86%) food samples, with the metacommunity defined as bacteria that were found in different food matrices. We also observed that closely related species with high abundance and uneven distribution across different sites could potentially exaggerate the dissimilarity between phylogenetically similar communities if they were measured using traditional species-based metrics (*P*_adj._ = 0.003), whereas this effect was mitigated by incorporating phylogeny (*P*_adj._ = 1). In summary, our tool can provide additional insights into microbial communities of interest and facilitate the use of phylogeny-based approaches in metagenomic analyses.

## INTRODUCTION

The field of microbial community studies has exploded in part due to technical advances in high-throughput sequencing and post-sequencing bioinformatics. It has also greatly expanded because of the observed importance of microbial activities to a wide array of biological processes, including but not limited to human health (e.g., human microbiome) (1–10), agricultural safety and management (11–13), and environmental diversity and conservation (14–20).

The importance of microbial community diversity to each of those processes is matched by the importance of correctly describing and quantifying the diversity in samples collected from these environments. Much work has focused on the taxonomic classification of DNA sequence data derived from the samples, with many software applications providing census data detailing the identity and abundance of species therein (21–24). However, comparatively little work has been done to improve how census data can be used to describe and compare diversity among samples, even though such comparisons underpin hypothesis testing related to the effects of the microbiome in these environments [25].

**FIG 1:**
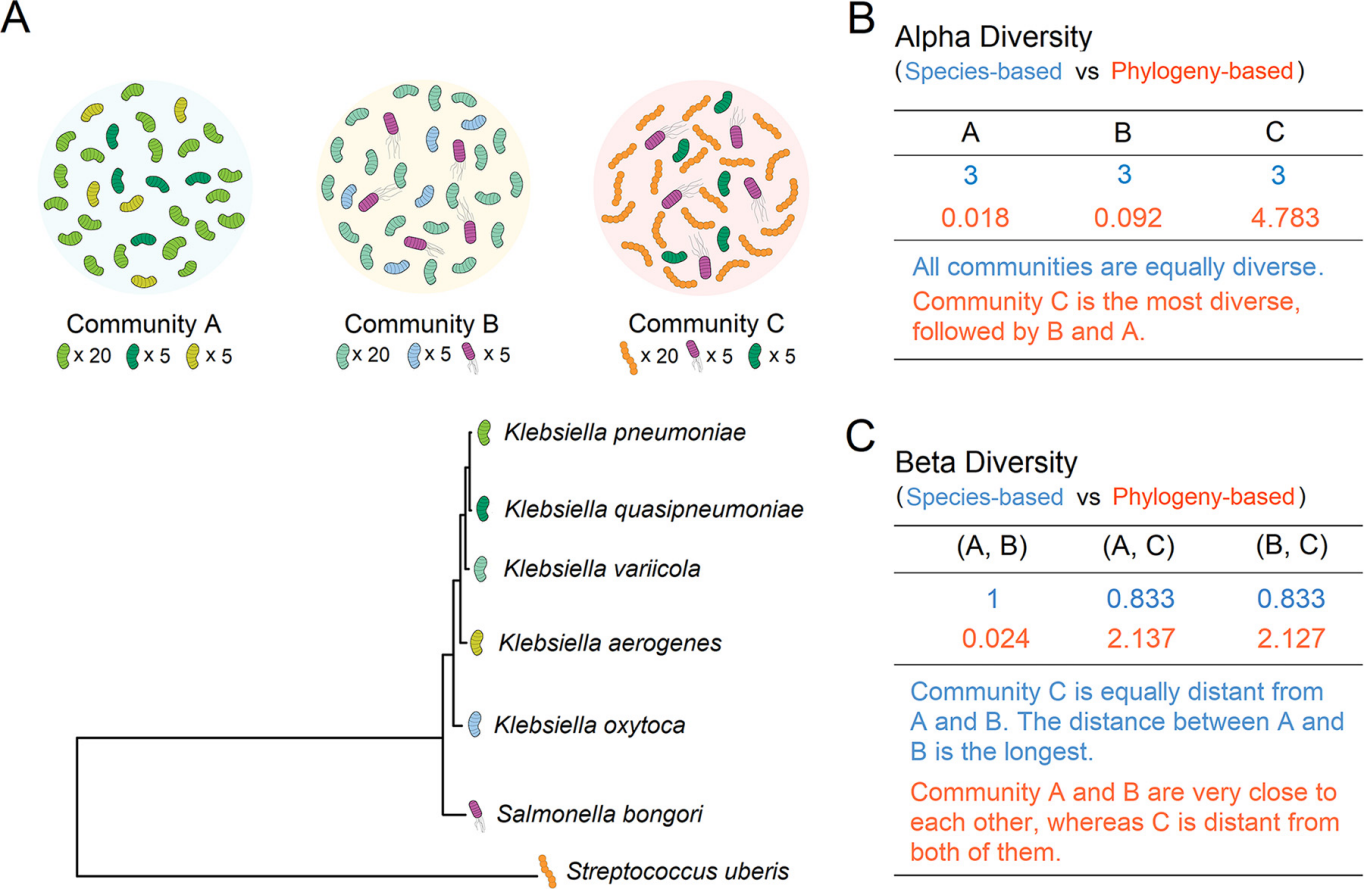
Alpha and beta diversity of three mock communities. (A) Three mock communities containing different bacterial species, with a phylogenetic tree showing their evolutionary relatedness. Species in community A share the same genus, species in community B share the same family, and species in community C share the same superkingdom. (B) Measuring the alpha diversity within these mock communities: example diversity metrics are species richness (species-based) and weighted Faith’s index (phylogeny-based). (C) Measuring the beta diversity between these mock communities: example diversity metrics are weighted Bray-Curtis (species-based) and weighted UniFrac (phylogeny-based). Whether or not phylogeny is incorporated into diversity analysis can lead to different conclusions.

The most widely used metrics describing and comparing different communities are based on information statistic indices, such as Shannon and Simpson’s indices for alpha diversity and Bray-Curtis and Jaccard distances for beta diversity. These methods consider a community as a list of species and their relative abundances. The number and evenness of species within a sample define alpha diversity, while overlap in species membership between different samples is used to quantify beta diversity. However, these species-based methods implicitly assume that all species are evolutionarily independent and ecologically equivalent, therefore conveying limited information regarding the function and evolutionary history of species (26, 27), which may lead to misleading conclusions regarding the diversity and differentiation among communities (Fig. 1). Given the fact that evolutionary relationships are not equivalent or irrelevant among different species, especially when quantifying differences among highly diverse communities, metrics incorporating phylogenetic information have been proposed, such as Faith’s index and UniFrac distance (28, 29). However, even though incorporating evolutionary information can aid in comparative community studies, phylogenetic metrics are still underutilized in this field (2–5, 13–19, 30, 31). This lack of use is partially due to the difficulty of constructing a robust molecular phylogeny for the diverse set of microbes found within natural metagenomic communities.

Many of the published metagenomic studies which have used phylogeny have done so by using phylogenies inferred from partial or full-length single gene sequences (e.g., the 16S rRNA gene) recovered from amplicon-based sequencing data (12, 20, 32–36). These phylogenies may be poorly resolved given the limited genetic data used and are constrained to only the set of taxa found in the samples, which limits the power of simulation modeling to describe community structure. Likewise, the construction of a molecular phylogeny based on shotgun sequencing data can be challenging due to the extreme diversity in gene content among different species, especially for a comprehensive phylogeny which extends the phylogenetic background (i.e., inclusion of species that can occupy the same niche but are not observed in the sample due to microbial competition) and allows flexibility in null model construction and hypothesis testing. Furthermore, to make use of a phylogeny, it must be congruent with the taxonomic output from the metagenomic classifier such that all identified species are represented in the phylogeny. The discontinuity between the research groups developing metagenomic taxonomic classification tools and those exploring community diversity means that phylogenetic tools to better explore and quantify microbial communities are sorely lacking.

In response to these problems, we have developed PhyloPlus, a web-based, freely available tool that allows users to generate custom phylogenies which incorporate all bacterial or archaeal species present within their metagenomic communities, enabling robust quantitative assessment of microbial community structure and diversity. Users need only upload a text file with a list of the species they wish to include, whereupon they are appended to a molecular phylogeny inferred from concatenation of multiple ubiquitous single-copy protein sequences (37), such that the resulting phylogeny contains all species in that published study as well as all user-defined taxa. In this paper, we demonstrate how phylogeny can and should be incorporated into metagenomic data analysis. We provide example analyses which demonstrate the power of incorporating phylogeny into metagenomic analysis through the use of this web-based tool and the outputs of two different taxonomic classifiers and by comparing diversity and structure within and between metagenomes from fermented food and human microbiome samples respectively. Incorporation of such phylogenies helped us to fully utilize all possible classification outputs generated by taxonomic classifiers using NCBI taxonomies, to provide additional insights into community structure, and to allow the use of phylogeny-based metrics and construction of null models; this further improved hypothesis testing in terms of *P* value inferences and prevented erroneous conclusions by minimizing potential inflation of type I errors.

## RESULTS

### Modified phylogenies contained an expanded range of bacterial species.

We added taxa corresponding to those present in the taxonomic classification outputs to the Genome Taxonomy Database (GTDB) reference bacterial phylogeny, and we referred to them as our expanded phylogenies. The expanded phylogenies were generated by first retrieving complete taxonomic lineages for genome assemblies present in the GTDB phylogeny using the NCBI taxonomic system, then inferring the location of additional species which were found only in the taxonomic classifier sources based on lineage information.

**FIG 2:**
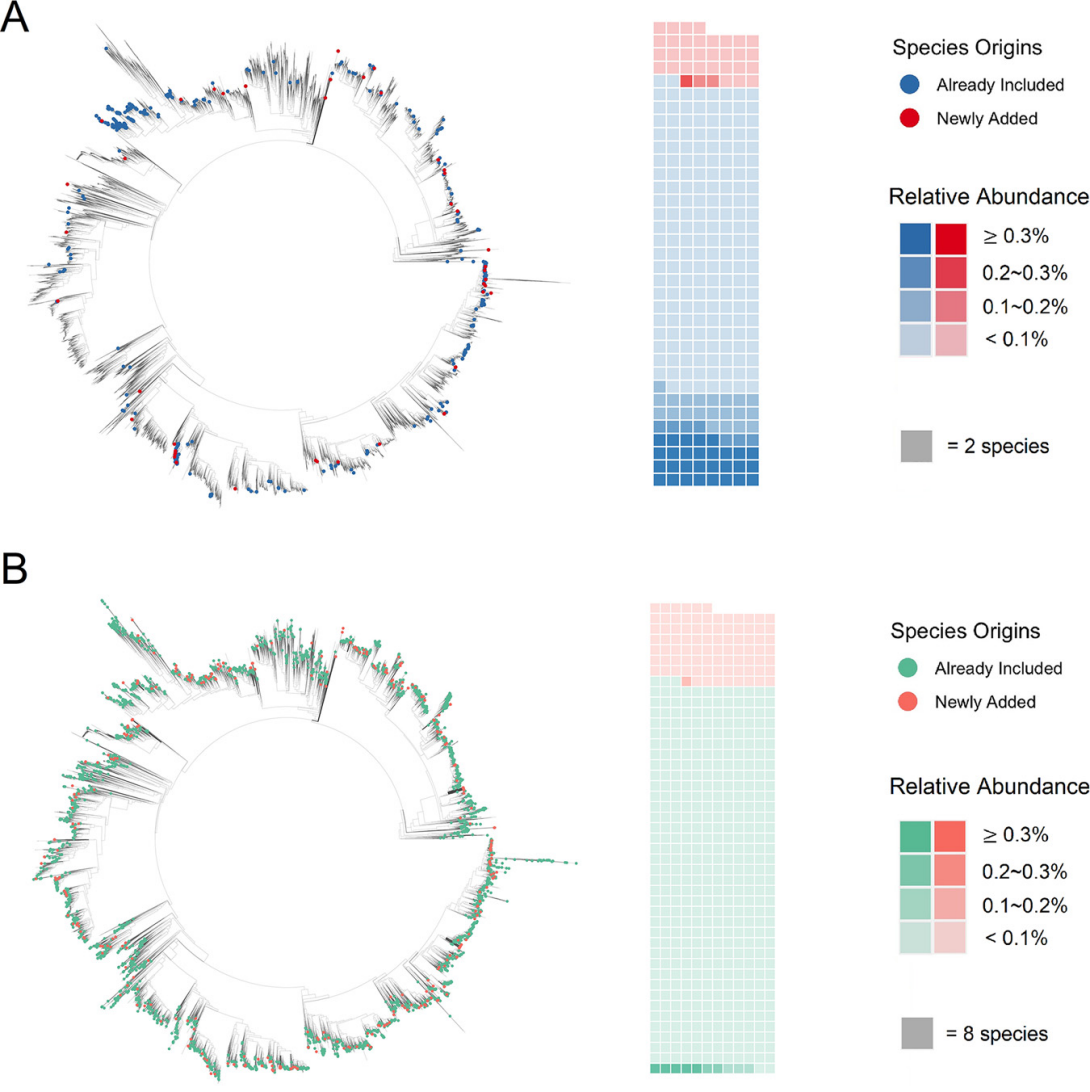
Insertion places and overall relative abundance for identified bacterial species. (A) Species identified in fermented food samples. (B) Species identified in human microbiome samples. In the final expanded phylogeny, the identified bacterial species could have already been included in the original phylogeny or have been newly added, and they are marked with different colors accordingly.

A total of 555 and 4,320 unique bacterial species were found in fermented food and human microbiome samples, respectively. The expanded phylogeny for fermented food samples contained an additional 69 bacterial species compared to the original GTDB phylogeny, i.e., species which were found in the taxonomic classifier reports but were missing from the original phylogeny. Likewise, the expanded phylogeny for human microbiome samples contained an additional 708 bacterial species. Insertion of these additional species increased the total branch length in the final expanded phylogeny by 0.91% (1974.086 to 1992.073) and 7.94% (1947.765 to 2102.475) for fermented food samples and human microbiome samples, respectively. Some of the newly inserted tips represent species which had relatively high abundance in the samples and were crucial for community diversity analysis (Fig. 2).

### Example analysis I: fermented food samples—alpha diversity and inference of community dispersion patterns.

Overall, bacterial communities in fermented foods were dominated by the phyla Proteobacteria and Firmicutes. The most abundant genera identified in fermented foods with dairy substrate were *Lactococcus* (48.51%), followed by Streptococcus (30.36%) and Escherichia (6.74%), whereas food samples with brine as the substrate were dominated by *Lactiplantibacillus* (17.16%), *Clostridioides* (13.08%), and *Levilactobacillus* (8.49%); and food samples with sugar substrate were dominated by *Komagataeibacter* (10.66%), *Liquorilactobacillus* (10.27%), and *Acetobacter* (9.63%). The compositional descriptions at other taxonomic scales regarding these foods, as well as three samples not belonging to these three categories, can be found in the original published study (31).

**FIG 3:**
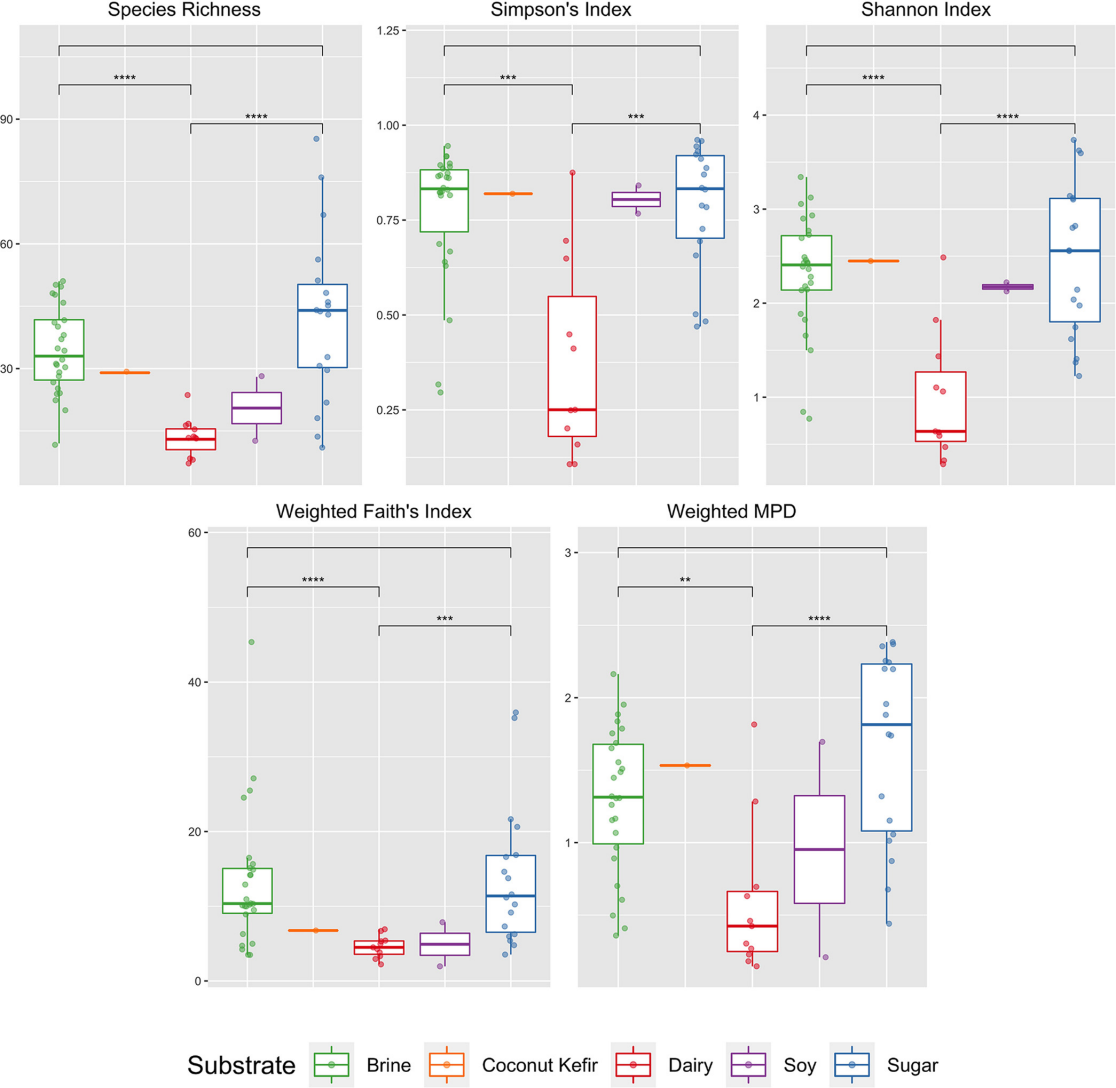
Alpha diversity for fermented food by different substrates. First row presents species-based metrics that were also used in the original study; second row presents phylogeny-based metrics. Pairwise *t* tests were performed to determine whether these alpha diversity metrics differed significantly between different substrate types; significant differences are shown by asterisks. *, *P ≤ *0.05; **, *P ≤ *0.01; ***, *P ≤ *0.001; ****, *P ≤ *0.0001. Coconut kefir and soy food had insufficient sample sizes for pairwise comparisons.

The original study utilized several species-based alpha diversity metrics to evaluate the community composition, including species richness, Shannon index, and Simpson’s index. We reproduced their analysis using the same species-based metrics and, as a contrast, additionally implemented phylogeny-based metrics which utilized evolutionary distances (i.e., branch length in the phylogeny), such as weighted Faith’s index and weighted mean pairwise distance (MPD). The expanded bacterial phylogeny was incorporated in the calculation of the phylogeny-based metrics. Pairwise *t* tests suggested that the phylogeny-based indicators weighted Faith’s index (dairy: 4.58 ± 1.48; brine: 13.20 ± 9.12; sugar: 13.90 ± 9.51) and weighted MPD (dairy: 0.59 ± 0.52; brine: 1.30 ± 0.50; sugar: 1.66 ± 0.65) were also capable of differentiating bacterial communities in dairy samples from communities in foods with brine or sugar as the substrate (Fig. 3).

Utilizing phylogeny-based metrics also allowed for the comparison of these metrics with a null model of community assembly, which has been frequently used in inference of community structure. In our example analysis, we further constructed such null models with the metacommunity defined as a pool of bacterial species that could be found in food matrices. During each permutation, tip labels of species belonging to the defined metacommunity were randomly shuffled and the phylogeny-based metric was recalculated based on the randomized placement of tip labels within the phylogeny. The resulting metric would represent a bacterial community where evolutionary relatedness between different species was randomly assigned, and the percentile where the observed values ranked among the series of permuted values would suggest community dispersion patterns. For example, a significantly lower observed value would suggest phylogenetic under-dispersion (clustering) where member species were closely related, and a significantly higher observed value would suggest phylogenetic over-dispersion.

**FIG 4:**
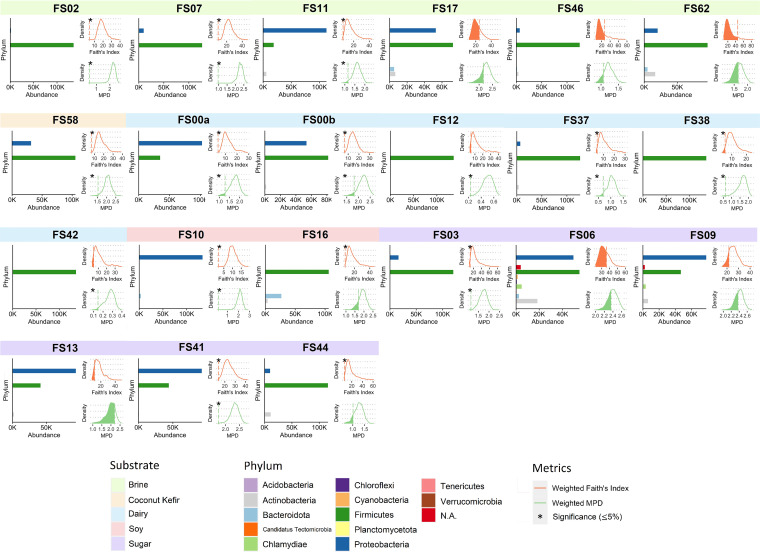
Phylum-level taxonomic composition and alpha diversity null distributions for selected fermented food samples. For each subplot: left, relative abundance of different phyla; upper right, null distribution of weighted Faith’s index; lower right, null distribution of weighted mean pairwise distance (MPD). Null distributions were generated by random shuffling phylogenetic tip labels; area under the curve shows the percentile in which the observed value ranked among the permuted values. Asterisks indicate that the percentile was less than or equal to 5%, suggesting phylogenetic under-dispersion of the bacterial community.

Most of the fermented food samples (44 out of 58, 75.86%) had significantly lower observed values compared to the permuted values, suggesting phylogenetic under-dispersion of these bacterial communities. The bacterial communities in the remaining food samples did not show signs of phylogenetic under-dispersion: some could be distinguished by their much more diverse phylum-level distributions (e.g., FS06), whereas the others might have similar phylum-level profiles as other under-dispersed communities, suggesting more complexity within the same phylum (e.g., FS13) (Fig. 4, Fig. S1).

10.1128/mbio.03455-22.3 FIG S1 Phylum-level taxonomic composition and alpha diversity null distributions for all fermented food samples. For each subplot: left, relative abundance of different phyla; upper right, null distribution of weighted Faith’s index; lower right: null distribution of weighted mean pairwise distance. Null distributions were generated by random shuffling phylogenetic tip labels, area under the curve represented the percentile where the observed value ranked among permuted values. An asterisk (*) indicates that the percentile was less than or equal to 5%, suggesting phylogenetic under-dispersion of the bacterial community. Download FIG S1, TIF file, 1.7 MB. Copyright © 2023 Huang et al. 2023 Huang et al. https://creativecommons.org/licenses/by/4.0/ This content is distributed under the terms of the Creative Commons Attribution 4.0 International license.

### Example analysis II: human microbiome samples—beta diversity and improved PERMANOVA analysis.

**FIG 5:**
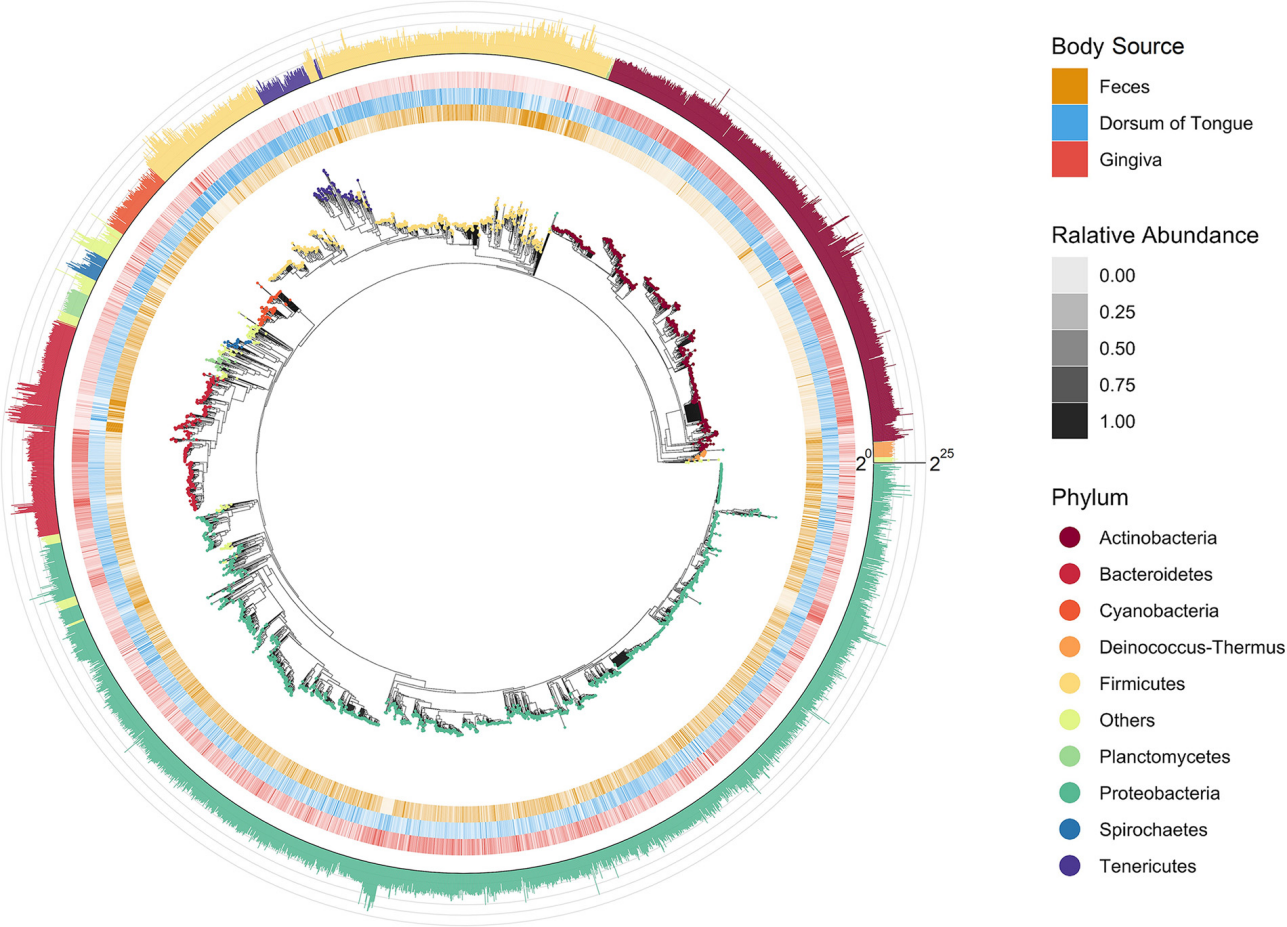
Bacterial diversity in human microbiome communities used in this study. Center: phylogenetic tree for bacterial species that were present in the communities, extracted from the expanded phylogeny. The tips are colored according to their phylum. Middle ring: relative abundance of a species compared across different body sources, color opacity represents the percentage of the species identified in each source. Outer ring: bar plot showing the total abundance of each species. Abundance data have been transformed into log_2_ scale, and bars are colored by phylum.

For an example of beta diversity analysis, bacterial communities were extracted from the publicly available Human Microbiome Project (HMP) representing 10 male and 10 female subjects and three body sources: feces, gingiva, and dorsum of the tongue. Overall, the microbiome from these three body sources was dominated by the phyla Bacteroidetes (36.48%), Actinobacteria (23.84%), Firmicutes (19.79%), and Proteobacteria (16.62%) (Fig. 5). Compared to communities in gingiva and dorsum of tongue samples, fecal communities showed a different pattern of taxonomic composition in which a few genera dominated: *Bacteroides* constituted 36.63% of the total abundance and *Phocaeicola* constituted 29.32%. In contrast, bacterial communities in dorsum of tongue (*Prevotella*: 14.57%; *Schaalia*: 14.17%; and Streptococcus: 12.75%) and gingiva samples (*Actinomyces*: 19.24%; *Neisseria*:15.77%; and *Corynebacterium*: 13.09%) had a more even distribution at the genus level.

**FIG 6:**
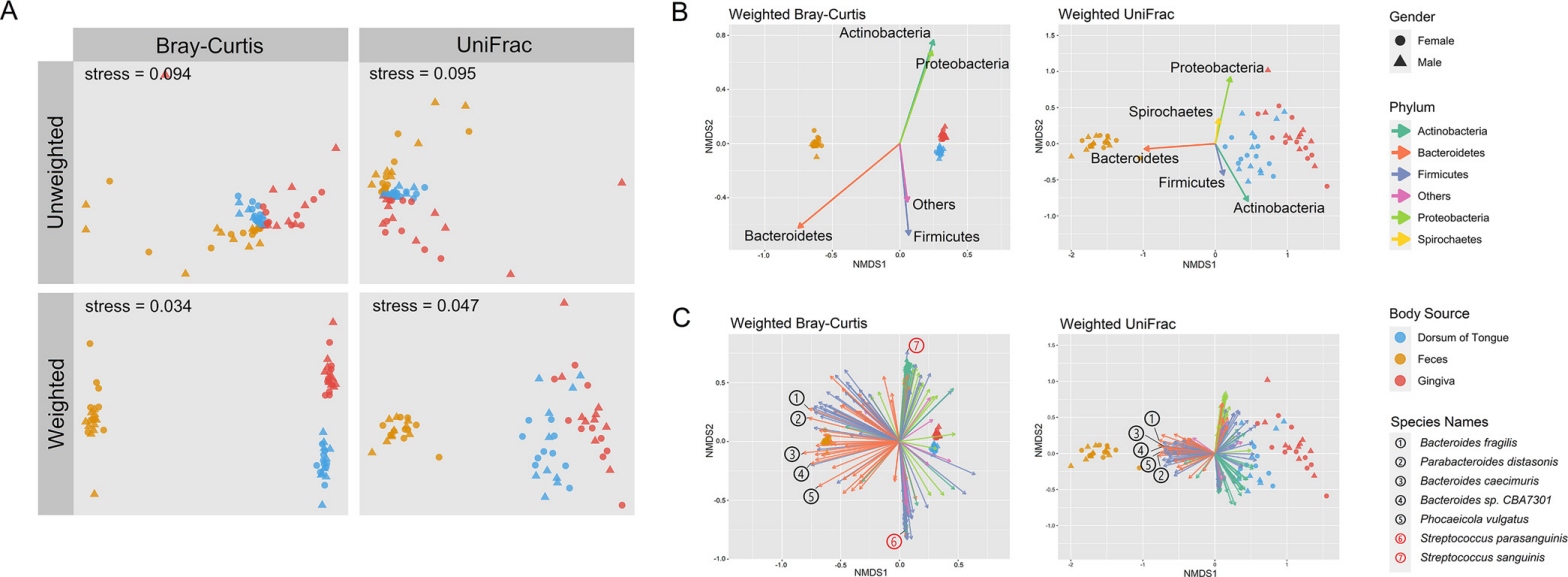
Beta diversity nonmetric multidimensional scaling (NMDS) plots. (A) NMDS plots showing separation among communities from different body sources using different beta diversity metrics. (B and C) Results of fitting significant (*P ≤ *0.05) taxon abundance vectors (phylum and species, respectively) on weighted Bray-Curtis and weighted UniFrac NMDS plots, rescaled so that the vector length equals the corresponding correlation coefficient (*R*). Only species with an abundance of ≥50,000 are plotted in panel C. Black numbers represent example species belonging to phylum Bacteroidetes which show strong correlation (*R* > 0.7) in both subplots; red numbers represent example species which only show strong correlation when weighted Bray-Curtis distances are applied.

Abundance-weighted metrics outperformed their unweighted counterparts in representing the distances among samples in the reduced two-dimensional space, with comparatively lower ordination stress (i.e., data distortion) values. Distinction between communities from different body sources was also more obvious when species abundance information was incorporated into the computation (Fig. 6A).

Fitting taxa abundance vectors onto the weighted Bray-Curtis and weighted UniFrac nonmetric multidimensional scaling (NMDS) plots further revealed that species within the phylum Bacteroidetes (e.g., Bacteroides caecimuris) constituted key determining factors that helped to discriminate fecal communities from the other two (Fig. 6B and C), regardless of which metric was used. Some closely related species which had high abundance and uneven distribution across dorsum of tongue and gingiva, for example, Streptococcus sanguinis (145,661 in gingiva and 4,930 in dorsum of tongue) and Streptococcus parasanguinis (475 in gingiva and 51,095 in dorsum of tongue), were key factors for distinguishing communities sampled from these two sites if they were measured using weighted Bray-Curtis, whereas the discriminating power was minimized by their close phylogenetic distances and therefore these species became weaker indicators if the distances were measured by weighted UniFrac.

Free permutation-based permutational multivariate analysis of variance (PERMANOVA) results suggested strong significance for all beta diversity metrics used in our study, as well as all pairwise comparisons in the *post hoc* analysis. However, tip shuffling-based PERMANOVA results suggested that we should not reject the null hypothesis that the centroids and dispersion of different groups of bacterial communities were equivalent when using unweighted UniFrac distance matrix (*P* = 0.180; Table 1). Additionally, not all pairwise *post hoc* analyses for weighted UniFrac gave significant results (*P*_adj._ = 1 for comparison between dorsum of tongue and gingiva communities, Table 2). This demonstrated that species-based metrics and phylogeny-based metrics could lead to different conclusions.

**TABLE 1:** PERMANOVA results for different beta diversity metrics

β-diversity metric	Category	Df	Sum of squares	Mean square	*R* ^2^	*F*-ratio	*P* ^*a*^	Significance^*b*^
Bray-Curtis								
Weighted	Body source	2	12.595	6.298	0.629	48.335	0.001	***
	Residuals	57	7.426	0.130	0.371			
	Total	59	20.022		1.000			
Unweighted	Body source	2	0.616	0.308	0.251	9.560	0.001	***
	Residuals	57	1.835	0.032	0.749			
	Total	59	2.451		1.000			
UniFrac								
Weighted	Body source	2	107.285	53.642	0.789	106.310	0.001/0.001	***/***
	Residuals	57	28.762	0.505	0.211			
	Total	59	136.047		1.000			
Unweighted	Body source	2	0.835	0.418	0.224	8.206	0.001/0.180	***/NS
	Residuals	57	2.901	0.051	0.776			
	Total	59	3.736		1.000			

a For Bray-Curtis dissimilarity where phylogeny was not applied in the computation, results are only for free permutations using the vegan:adonis function. For phylogeny-based UniFrac metrics, results for both adonis free permutation and tip shuffling-based permutation are included and are shown in the form of vegan:adonis result/tip shuffling result.

b Statistical significance is indicated by different marks. NS, not significant; *, *P* ≤ 0.05; **, *P* ≤ 0.01; ***, *P* ≤ 0.001.

**TABLE 2:** Pairwise *post hoc* PERMANOVA results for different beta diversity metrics

β-diversity metric	Pair^*a*^	*F*-ratio	*R* ^2^	*P* ^*b*^	Adj. *P*^*c*^	Significance^*d*^
Bray-Curtis						
Weighted	F vs D	54.492	0.589	0.001	0.003	**
	F vs G	46.866	0.552	0.001	0.003	**
	D vs G	43.080	0.531	0.001	0.003	**
Unweighted	F vs D	10.047	0.209	0.001	0.003	**
	F vs G	9.813	0.205	0.001	0.003	**
	D vs G	8.625	0.185	0.001	0.003	**
UniFrac						
Weighted	F vs D	156.938	0.805	0.001/0.001	0.003/0.003	**/**
	F vs G	171.714	0.819	0.001/0.001	0.003/0.003	**/**
	D vs G	21.015	0.356	0.001/0.986	0.003/1	**/NS
Unweighted	F vs D	8.654	0.185	0.001/-	0.003/-	**/–
	F vs G	7.944	0.173	0.001/-	0.003/-	**/–
	D vs G	8.182	0.177	0.001/-	0.003/-	**/–

a F, feces communities; D, dorsum of tongue communities; G, gingiva communities.

b For Bray-Curtis dissimilarity where phylogeny was not applied in the computation, the results are only for free permutations using the vegan:adonis function. For phylogeny-based UniFrac metrics, results for both adonis free permutation and tip shuffling-based permutation are included and are given in the form of vegan:adonis result/tip shuffling result.

c *P* values were adjusted using the Holm method.

d Statistical significance is assigned according to adjusted *P* value. –, *post hoc* analysis not performed; NS, not significant; *, *P *≤* *0.05; **, *P *≤* *0.01; ***, *P *≤* *0.001.

## DISCUSSION

The emergence of metagenomics as an accessible tool for exploring biological communities has required three elements to realize its potential, including (i) high-throughput sequencing, (ii) high-performance software to assemble or classify sequence data and estimate the taxonomic abundances (21–23), and (iii) appropriate methods for use in comparing the biological communities inferred therein, the last of which is the scope of this study. Species-based metrics such as Shannon index and Bray-Curtis and Jaccard distances readily describe the variability within and differentiation between biological communities and are widely used in metagenomics. However, these metrics, and their relatives which treat each member of a community as an equal, wholly independent unit, fail to incorporate the fact that members of a community of biological organisms may differ widely in their evolutionary relatedness. As such, the relatedness of individuals within a community can profoundly affect our inference of the diversity and relative similarity of those communities (Fig. 1).

Data analysis metrics which incorporate phylogeny into inferences of biological diversity exist and have been successfully employed to describe different communities (28, 29). Yet despite the theoretical advantages posited by the incorporation of phylogeny, its use in microbial metagenomics is still limited (2–5, 13–19, 30, 31). This underutilization of phylogeny arises in large part due to the difficulty of constructing a comprehensive microbial phylogeny in which all members of a diverse community are represented. This limitation is compounded by the fact that many phylogeny-based diversity analysis tools require all identified taxa within the communities to be present on phylogenetic tree tip labels (38). Thus, if the available phylogeny does not include all species present in the community sample, the analysis may be biased through exclusion of these species, or it may be inoperable. By removing records containing taxa outside the phylogeny from the original community file, researchers can force an output, but they do so without full utilization of the taxonomic classification software. Alternatively, studies that seek to construct a phylogeny from single gene sequences recovered from amplicon sequencing data lack the broader phylogenetic background (i.e., species beyond those which are present in the sample under study) and as such cannot fully utilize null models derived from randomization of the phylogeny to engage in hypothesis testing. Thus, limitations in the availability and completeness of phylogeny restrain its use in the quantitative description of microbial metagenomic analyses.

In response to this, we developed PhyloPlus, a publicly available web portal that can generate user-defined phylogenies. The portal works by extracting a user-defined set of species labels (via their NCBI taxonomy IDs) from the GTDB molecular bacterial or archaeal phylogeny, or by appending them to the original phylogeny based on their taxonomic lineage information if they are not already present. In the current study, we extracted all taxonomic labels from metagenomic classification reports on a data set of fermented food samples using the Kaiju software, as well as reports on another data set of human microbiome samples using the Kraken2 software. We then appended taxonomic labels from each data set to the GTDB bacterial phylogeny, respectively, for use in our example diversity analyses. The expanded phylogenies generated by our portal could be applied to downstream diversity analysis seamlessly after taxonomic classification by classifiers utilizing NCBI taxonomies, such as Kraken2 and Kaiju software, and its broad phylogenetic background also allowed flexibility in null model construction and hypothesis testing.

To demonstrate how incorporation of phylogeny dramatically improved our inference of metagenomic communities, we contrasted species-based and phylogeny-based diversity metrics as applied to two different groups of published high-quality data sets. Specifically, we contrasted the inferred community diversity within and among samples (alpha and beta diversity) using both approaches, as well as comparing inference of statistical significance among samples. Lastly, we discussed how the use of phylogeny can provide more accurate differentiation among groups where traditional species-based metrics would fail to distinguish communities or worse, wrongly infer patterns based on the assumption that all members are equivalent; and how the inclusion of phylogenetic relatedness could enable explicit hypothesis testing, where the phylogenetic structure observed within samples may serve as constructive null-models for indication of different biological processes.

The performance of a diversity measure was defined as the combined result of its conceptual basis and the observed composition of the communities being studied. For the fermented food data set, samples with the dairy substrate were characterized by the presence of two dominant genera, *Lactococcus* and Streptococcus. Species in these two genera were phylogenetically similar and highly abundant in dairy food communities, which led to significantly lower weighted Faith’s index and weighted MPD values compared to food samples of other substrate types that had more evenly distributed communities (Fig. 3). Comparison of phylogeny-based metrics with the species-based metrics used in the original study suggested similar discriminative power to differentiate dairy fermented food from food with brine or sugar substrate in terms of their alpha diversity. This could be explained by strong correlation observed between some diversity metrics (39), for example, the correlation between weighted MPD and Shannon index was because the former was the phylogenetic extension of the latter (40).

In addition to providing alternative metrics to describe alpha diversity, the use of phylogeny allows for the construction of null models which further provide a way to assess community structure. In an ecological context, community phylogenetic approaches are often used to assess the effect of environmental filtering (under-dispersion or clustering of closely related species) and competition (over-dispersion of distantly related species) (41), and often utilize comparisons of diversity metrics (e.g., percentile, standard effect size). Relevant studies have been done on both eukaryotic (34, 42–45) and prokaryotic (33, 46, 47) communities. In our study, even with a metacommunity defined as the pool of bacterial species which were able to grow in food matrices, we still observed that most of the bacterial communities in fermented food were under-dispersed. This could be largely explained by the specific environment formed during fermentation (e.g., acidic or alkaline food, or food with high salinity), and the addition of starter cultures, which favored the growth of a group of bacterial species that share similar traits. However, we still observed that the bacterial communities in some samples did not show signs of under-dispersion. For example, the water kefir sample (FS13), which had sugar as the substrate, had a taxonomic distribution in which only two phyla dominated. However, this food sample was unique for its high abundance of *Kluyvera* species, which were rarely identified or absent in other sugar fermented foods. The distinct phylogenetic locations of species belonging to the genus *Kluyvera* from other genera in the phylum Proteobacteria contributed to the result in which the observed alpha diversity values for this sample ranked closer to the center of the corresponding null distributions. This also suggested that bar plots at high taxonomic levels (e.g., phylum, class), which have been widely used to visualize taxonomic profiles of metagenomic samples (48, 49), might be uninformative or misleading if used without additional analysis; and that the incorporation of phylogeny-based null model construction helped capture this information.

The incorporation of phylogeny also enabled alternative approaches to construct null models which were used to test hypotheses regarding differences among different communities. Traditionally, to compare communities groupwise, permutation is performed by randomly shuffling the labels on the rows which identify each community as belonging to a particular group (50). While this approach is widely applicable (13, 14, 17, 32), shuffling grouping labels caused variation in aspects such as species richness and occupancy rates across communities. The less-constrained models, therefore, could cause potential inflation of type I errors (40). Tip shuffling within a phylogeny randomized only the evolutionary relatedness among different taxa while keeping all patterns in the community data untouched; it also allowed permutations on any phylogeny-based diversity metrics without additional mathematical analyses to generate the null model. For beta diversity analysis in our study, more conservative conclusions were drawn from null models based on tip shuffling methods (Table 1, Table 2). In the case of the beta diversity metric weighted UniFrac, the species S. sanguinis and S. parasanguinis exemplified how the incorporation of phylogeny-based metrics and tip shuffling-based null models could help to rectify exaggeration of distances between communities from dorsum of tongue and gingiva, compared to those measured using weighted Bray-Curtis (Fig. 6). Because both sampling sites were in the oral cavity, we would expect that communities in these two sites should be more functionally or phylogenetically convergent, or at least less distinct than suggested otherwise, and that the incorporation of a complete phylogeny would take us a step closer to the “true” representation of the communities.

We note that some phylogeny-based diversity metrics, such as mean nearest taxon distance, are highly sensitive to phylogenetic terminal topology, and therefore may be influenced by the addition of taxon labels to the phylogeny, where a set of added taxa may form a polytomy due to the lack of lineage information to infer their locations. This suggests that in the future, the application of rate-smoothing algorithms which use the existing molecular branch lengths to apply more natural distances among these taxa will be useful. We anticipate that continued development of molecular phylogenies and rate smoothing can be incorporated into our web portal to provide the most valuable phylogenetic reconstructions possible for widespread application in the quantitative description of microbial metagenomic communities (51, 52).

## MATERIALS AND METHODS

### Metagenomic data retrieval and preprocessing.

We retrieved publicly available metagenomic data from two different projects. (i) Raw metagenomic shotgun sequencing files of a total of 58 fermented food samples were retrieved from the European Nucleotide Archive database, as described in a recently published study (31). These samples represented fermented food of different substrate types: brine (*n* = 26), sugar (*n* = 18), dairy (*n* = 11), soy (*n* = 2), and coconut kefir (*n* = 1). Quality trimming was performed using Trimmomatic (v0.39; sliding window size: 4; average quality required: 20) (53). (ii) Human microbiome data were retrieved from the National Institutes of Health (NIH) HMP via its online portal (https://portal.hmpdacc.org). Healthy adult subjects between 18 and 40 years of age were recruited for that project and were sampled one to three times from 15 (male) and 18 (female) distinct body habitats (1). A total of 60 metagenomic shotgun sequencing files were downloaded for use in our study, including 10 male and 10 female subjects, each covering three body sources: feces, gingiva, and dorsum of tongue. All data downloaded via the portal were shotgun-sequenced using Illumina Genome AnalyzerIIx platform with a 101 bp, paired-end reads approach and had already been processed to mask or remove human host contaminants, duplicated reads, and low-quality reads (54). The corresponding metadata for sequencing files from both sources are listed in Supplemental Material Data Set S1.

### Metagenomic taxonomic classification and rarefaction.

For fermented food metagenomic files, we adopted the software and parameters from the original study to conduct the taxonomic classification: Kaiju (v1.9.0) was used for the taxonomic classification using the nonredundant protein database for bacteria, archaea, and viruses (constructed on 24 February 2021) (24). A threshold of 0.1% was used to exclude any species with a relative abundance lower than this value from the final report. An in-house Python script was used to retain only bacterial species in the final report for our own analysis.

For human microbiome data, Kraken2 was used to obtain the corresponding taxonomic profiles (21). The standard bacterial reference library which included RefSeq complete bacterial genomes was downloaded on 1 March 2021 and was used for the classification. Bracken was used to estimate the number of reads originating from each species present in a sample based on Kraken2 output files (55).

Rarefaction was performed for each bacterial community from both sources before any diversity analyses so that the total abundance was kept the same as the smallest community (185,369 and 5,533,226 classified bacterial species abundance for samples from fermented food and human microbiome, respectively).

### Phylogenetic tree expansion.

The GTDB bacterial phylogenetic tree was utilized in this project (37), and the latest release was downloaded via its publicly available server (https://data.gtdb.ecogenomic.org/releases/latest/).

The addition of species found in taxonomic classification reports to the GTDB phylogeny was achieved utilizing the taxonomic lineage of all tips present in the phylogeny. Complete lineage information (from species to superkingdom) was appended to the phylogenetic tips, as well as bacterial species identified in classification outputs. The taxonomic rank with which a given query species could be mapped to the phylogeny was determined by the lowest taxonomic rank possible in which at least one of the tips in the original GTDB bacterial phylogeny was shared with the query species. A subtree connecting all tips sharing the same taxonomy ID at the determined taxonomic rank was extracted; the insertion node for the query species was the root of the subtree and the branch length was the average distance of all children tips to the subtree root.

More detailed descriptions of methods to convert information from both the original phylogeny and taxonomic classification outputs into species names/IDs under the NCBI taxonomy system, compute the insertion node and corresponding branch length, detect and remove potential outlier tips after extraction of the subtree, and prune the tree so that each unique species is represented by a single tip can be found in Supplemental Material Text S1. A web tool (PhyloPlus) implementing the process we employed in this paper to add a user-defined set of species via their NCBI taxonomy IDs to GTDB bacterial/archaeal phylogenies is publicly available at http://phylo.jifsan.org.

### Alpha diversity analysis.

Alpha diversity analysis was conducted on fermented food metagenomic samples. All species-based alpha diversity measures mentioned in the original study were calculated using the vegan package in R (56), including species richness, Shannon index, and Simpson’s index. Additionally, the phylogeny-based metric weighted MPD was calculated using the picante R package (38), and an abundance-weighted version of Faith’s index was calculated using a self-written R function adopted from Swenson (40). Pairwise *t* tests were performed to determine whether significant differences were present between communities of different substrate types in terms of alpha diversity measurements.

### Beta diversity analysis.

Beta diversity analysis was conducted on human microbiome samples. The weighted and unweighted Bray-Curtis dissimilarity metrics were calculated using the vegan R package with the vegdist function. Weighted and unweighted UniFrac were calculated with the rbiom R package using the unifrac function [57]. The metaMDS function from vegan package was used for the NMDS which visualized comparisons among different communities, based on the distance matrices calculated above. The envfit function from vegan package was applied to fit phylum and species abundance vectors onto the weighted Bray-Curtis and weighted UniFrac NMDS plots to identify potential taxa which drove the separation pattern in each plot.

### Null model construction and statistical analysis.

The phylogeny-based alpha diversity metrics, weighted Faith’s index and weighted MPD, were used to infer community dispersion patterns via generating a null model based on randomizing the phylogeny. The function tipShuffle from picante R package was used to randomize the names of the taxa on the phylogeny, and the pool of individual taxa (metacommunity) for random shuffling was defined as the one containing all bacteria species which could be found in food matrices (i.e., the collection of bacteria species identified by the classification software for all fermented food samples).

PERMANOVA was used to investigate whether the overall composition of bacterial communities differed statistically by sampling site for human microbiome samples. Distance matrices generated using beta diversity metrics were analyzed using the adonis function from vegan R package with 999 free permutations. For UniFrac metrics in which the phylogeny was included in the calculation, a subtree containing all bacterial species identified in the human microbiome used in this study was extracted. Tip shuffling was applied to the subtree, UniFrac distance matrices were then recalculated for the same community data using different randomized phylogenies, and a pseudo *F*-ratio was calculated for each distance matrix (50). The *P* value was obtained based on 999 instances of random tip shuffling. For both types of permutation, a *post hoc* pairwise test was performed upon identification of significant results, and the *P* values were adjusted using the Holm method.

### Data availability.

The web portal is freely available at https://phylo.jifsan.org. Upon completion of the process, users will receive an email containing the download link for the output folder. The output folder contains (i) four newick tree files, including two phylogenies incorporating species from the user input source as well as nonredundant species present in the reference phylogeny (comprehensive_names.tree, comprehensive_taxIDs.tree), and two incorporating only species provided by users (user_names.tree and user_taxIDs.tree); (ii) one summary file for the insertion of species provided in the user input, detailing the taxonomic rank with which a species can be mapped to the reference phylogeny, its assigned branch length, and the number of reference tips used for the inference; and (iii) one text file recording any potential changes made to the user input species taxonomy IDs (e.g., outdated or merged taxIDs.).

The command line version of this process can be found at https://github.com/Dennis-xyHuang/PhyloPlus.

10.1128/mbio.03455-22.1 TEXT S1 Supplementary information. Download Text S1, PDF file, 2.2 MB. Copyright © 2023 Huang et al. 2023 Huang et al. https://creativecommons.org/licenses/by/4.0/ This content is distributed under the terms of the Creative Commons Attribution 4.0 International license.

10.1128/mbio.03455-22.2 DATA SET S1 Metadata of metagenomic sequencing files used in this study. Download Data Set S1, XLSX file, 0.02 MB. Copyright © 2023 Huang et al. 2023 Huang et al. https://creativecommons.org/licenses/by/4.0/ This content is distributed under the terms of the Creative Commons Attribution 4.0 International license.
