# Supplementary figures and images for: PhyloPlus: a Universal Tool for Phylogenetic Interrogation of Metagenomic Communities
Source: mBio. 2023 Jan 16;14(1):e03455-22. doi: 10.1128/mbio.03455-22 (PMC9973285; doi:10.1128/mbio.03455-22)

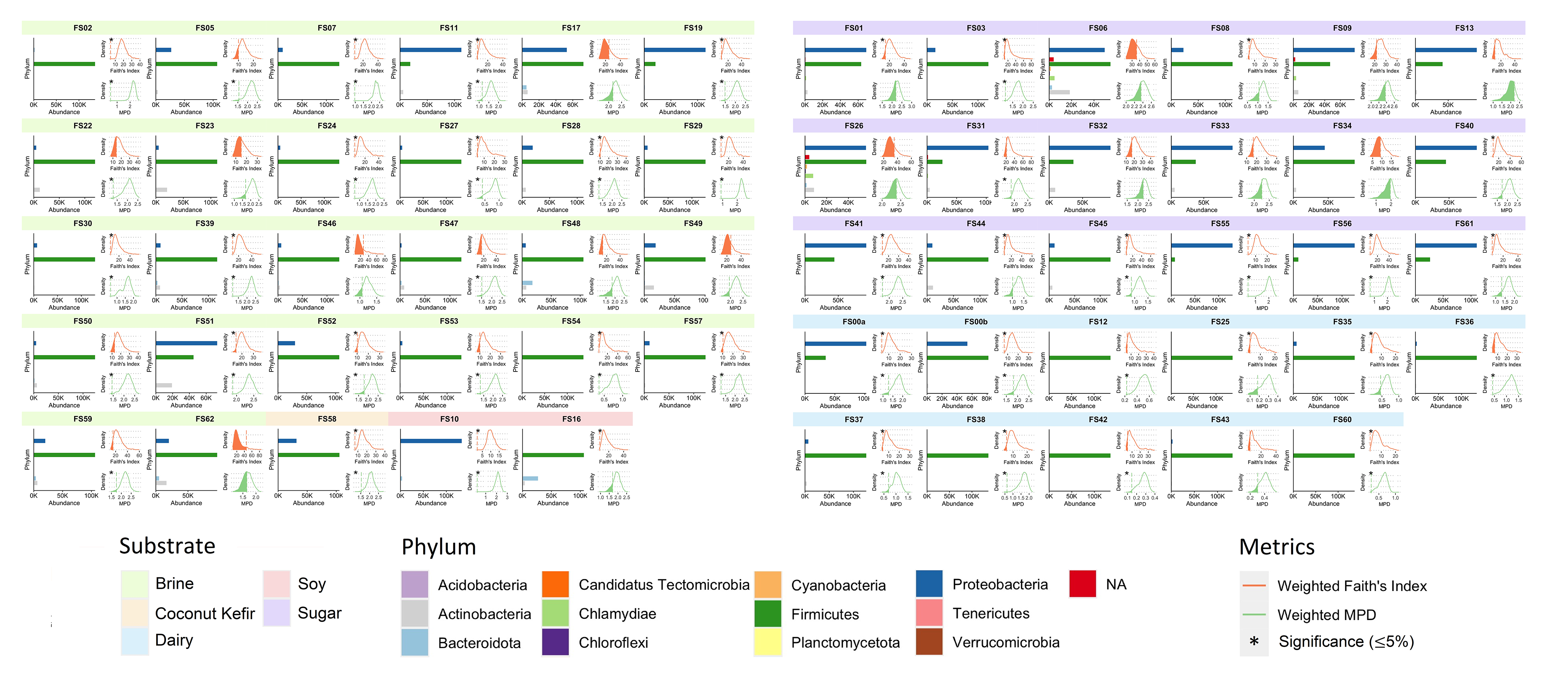

Supplement: FIG S1 [file mbio.03455-22-s0003.tif]
