# Supplementary material for: PhyloPlus: a Universal Tool for Phylogenetic Interrogation of Metagenomic Communities
Source: mBio. 2023 Jan 16;14(1):e03455-22. doi: 10.1128/mbio.03455-22 (PMC9973285; doi:10.1128/mbio.03455-22)
Supplement: TEXT S1 [file mbio.03455-22-s0001.pdf]

Supplementary Information for

PhyloPlus: A Universal Tool for Phylogenetic Interrogation of Metagenomic Communities

Supplementary Methods

The methods described below give detailed information regarding how the modification and expansion to the original Genome Taxonomy Database (GTDB) phylogeny was made in each step [1].

Determination of NCBI Taxonomy ID for Each Genome Assembly.

Tip labels in the original GTDB phylogenies represent genome assemblies using the National Center for Biotechnology Information (NCBI) GenBank or RefSeq accession numbers, prefixed by "GB_" or "RS_", respectively. Assembly accession numbers were collected by removing the prefix of each of the tip labels in the phylogeny. The accession numbers were then searched against the NCBI Assembly database to retrieve the most up-to-date NCBI taxonomy ID (taxID) assigned to each of the genome assemblies.

The taxIDs for all bacterial genome assemblies that were used to build the Kraken2 standard bacterial database on Mar 1st, 2021 were retrieved directly from the Kraken2 seqid2taxid.map file. Bacterial species from the Kraken2 source were used to determine default threshold values used for removal of potential outlier tips in the following steps (Fig. 1).

Full Lineage Information Extraction.

Each of the non-redundant taxIDs retrieved above was searched against the NCBI Taxonomy database to fetch its full lineage information. As for the retrieved full lineage information, taxIDs that corresponded to different taxonomic ranks were collected, including superkingdom, phylum, class, order, family, genus, species group, and species. The lineage information was recorded in three individual text files, representing full lineage for each of the tips present in the original phylogeny, for each of the non-redundant species-level taxIDs identified in the original phylogeny, and for each of the non-redundant species-level taxIDs provided via the user input, respectively. All searches against NCBI databases were processed in batch using the Biopython package [2].

Appending Species to the Original Phylogeny.

For each of the unique species identified in the user input and GTDB phylogeny, the sequence of taxIDs at different taxonomic ranks was searched against full lineage information appended to the tip labels, starting from the lowest rank (species) to the highest (superkingdom). This

determined the lowest taxonomic rank possible where at least one of the genome assemblies in the original GTDB phylogeny shared with the query species.

Upon determination of the taxonomic rank to map the query species, all tips within the original phylogeny that shared the same taxID at the corresponding rank were extracted (e.g., all tips sharing genus-level taxID 226 for locating species *Alteromonas* sp. 76-1, where the exact species was not found in the original phylogeny, therefore its location was inferred using its congeneric taxa), their most recent common ancestor (MRCA) node was identified using getMRCA function from the ape R package [3]. A subtree rooting at the MRCA node was extracted, and the average distance of all its children tips that shared the same taxID at the determined taxonomic rank to the MRCA node was calculated. Then the species label (taxID or scientific name) was appended to the original phylogeny using add.tips function from the phangorn R package with the MRCA node being the place to bind the tip and the computed average distance being the branch length [4].

Removal of Potential Outliers and Tree Pruning.

Taxonomic misclassification can result in extreme outlier tips that could cause the computed MRCA node to reside close to the base root of the phylogeny, leading to abnormally long branch length assigned to the query species. Therefore, removal of potential outlier reference tips was done during the process of determining the MRCA node and computing the branch length.

For each member in the group of reference tips that were used to map a particular query species, its average distance to other group members (d_{avg}) was calculated. Then the mean and standard deviation were calculated for all d_{avg} values within the reference group. Potential outliers were defined as tips whose d_{avg} value exceeds mean plus n times standard deviations (n = 1, 2, 3, 4, and 5, respectively). For species mapped at different taxonomic ranks, different values of n were applied where the finalized threshold values were determined by considering how much improvement has been made in terms of branch length distributions and how much phylogenetic information was retained after removal of potential outlier tips (Fig. 2 and Table 1).

For groups containing only two reference tips where detection of potential outlier tips based on mean and standard deviation was impractical, the fraction

$$\frac{distance\ to\ the\ MRCA\ node}{distance\ to\ the\ base\ root}$$

for the more distant tip was used to indicate if an outlier was present in the reference group (threshold used in this case: fraction ≥ 0.75). The potential outlier tip was determined based on the lowest taxonomic rank it shared with its neighboring tips, where two nodes back were taken to extract the corresponding subtree. The one with a comparatively higher-level taxonomic rank shared with the neighboring tips was determined to be the outlier.

Lastly, the phylogeny was pruned by removing all original tip labels which represented a single genome assembly, so that all inter-tip distances within the modified output phylogeny represented interspecific distances under NCBI taxonomic system.

Supplementary Notes

The taxa *Isorropodon fossajaponicum* symbiont (taxID 883811) and *Abyssogena phaseoliformis* symbiont (taxID 596095) were identified in the Kraken2 standard bacterial library and were assigned taxonomic rank species by NCBI, but were not added to the modified phylogeny, as they can only be mapped at the superkingdom level where the inference of their location using the entire bacterial phylogeny was computationally infeasible and biologically meaningless.

Updates and merging of NCBI taxIDs are continuous, for example, during the time of writing this manuscript, taxIDs 147467 and 861208 that were retrieved from the Kraken2 source have been merged into taxIDs 1296 and 1183401, respectively. These updates can cause fatal errors for phylogenetic tree parsers when the corresponding species scientific names were used as tip labels, due to the fact that multiple tips share identical labels.

To handle the above two cases, some of the taxIDs uploaded by the user to the web portal may be (1) deleted prior to processing if they belong to the wrong superkingdom (e.g., adding an archaeal species to the bacterial phylogeny) or can only be mapped at the superkingdom level; or (2) modified or deleted if they are updated or merged into existing taxIDs, respectively. Any changes to the user input are recorded in the note.txt file, and the user may need to modify the taxIDs present in the metagenomic classification reports accordingly to allow full compatibility between the classification outputs and the phylogeny.

During insertion of each of the non-redundant species-level taxIDs identified in the original phylogeny, not all species names are mapped back into the original phylogeny, as some of them may represent "pseudo" species-level names (e.g., Enterobacteriaceae bacterium, taxID: 1849603, indeed represents a family but this taxID is assigned the rank species by NCBI and was included in the Kraken2 standard bacterial library). A filter for at least having a recorded species- and genus-level taxID in the full lineage was applied and only the filtered species were inserted back to the original phylogeny.

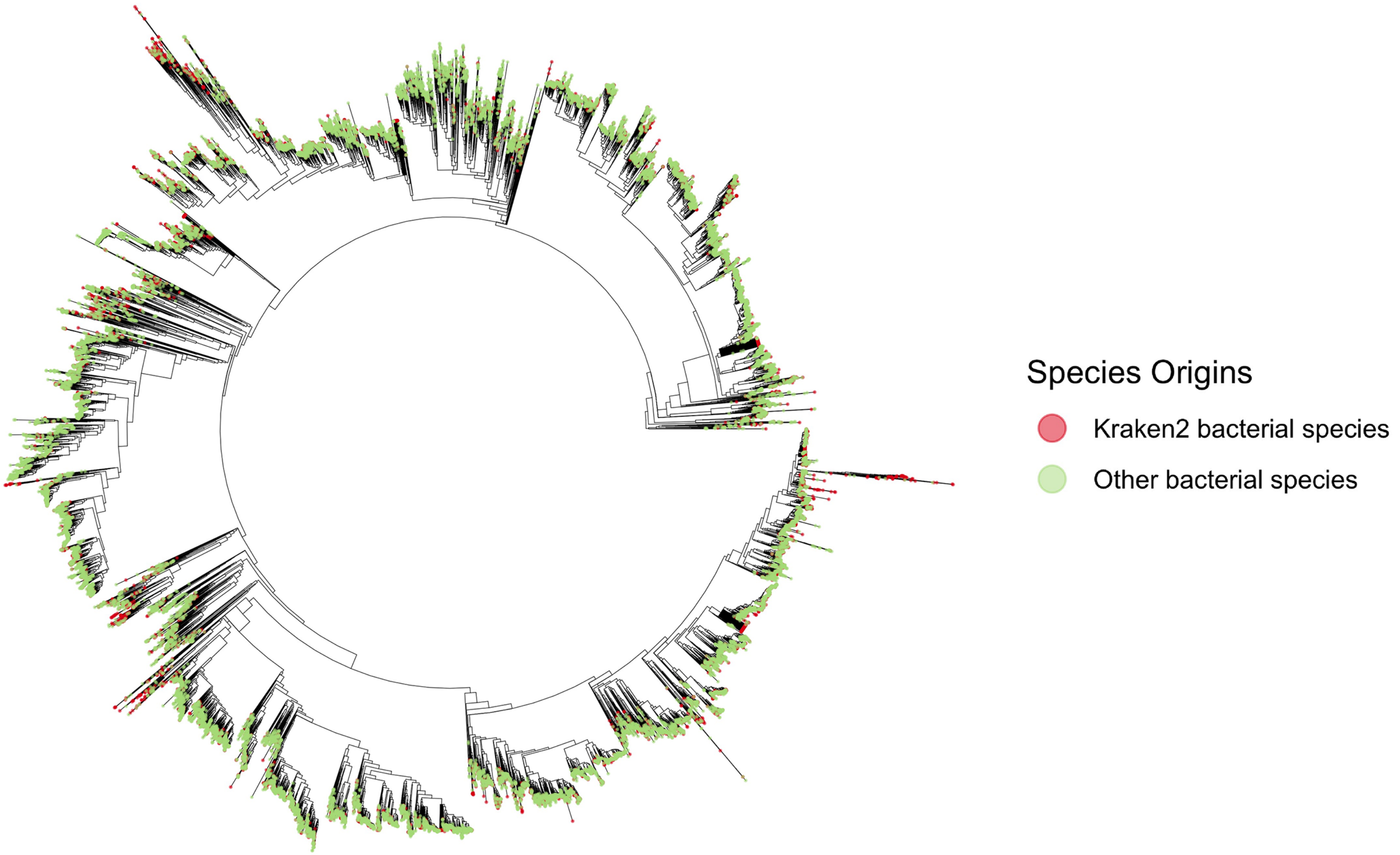

Fig. 1. Insertion places for 6,416 bacterial species identified in Kraken2 standard bacterial reference library (downloaded on Mar 1st, 2021) to the original phylogeny. These bacterial species were distributed across the entire phylogeny.

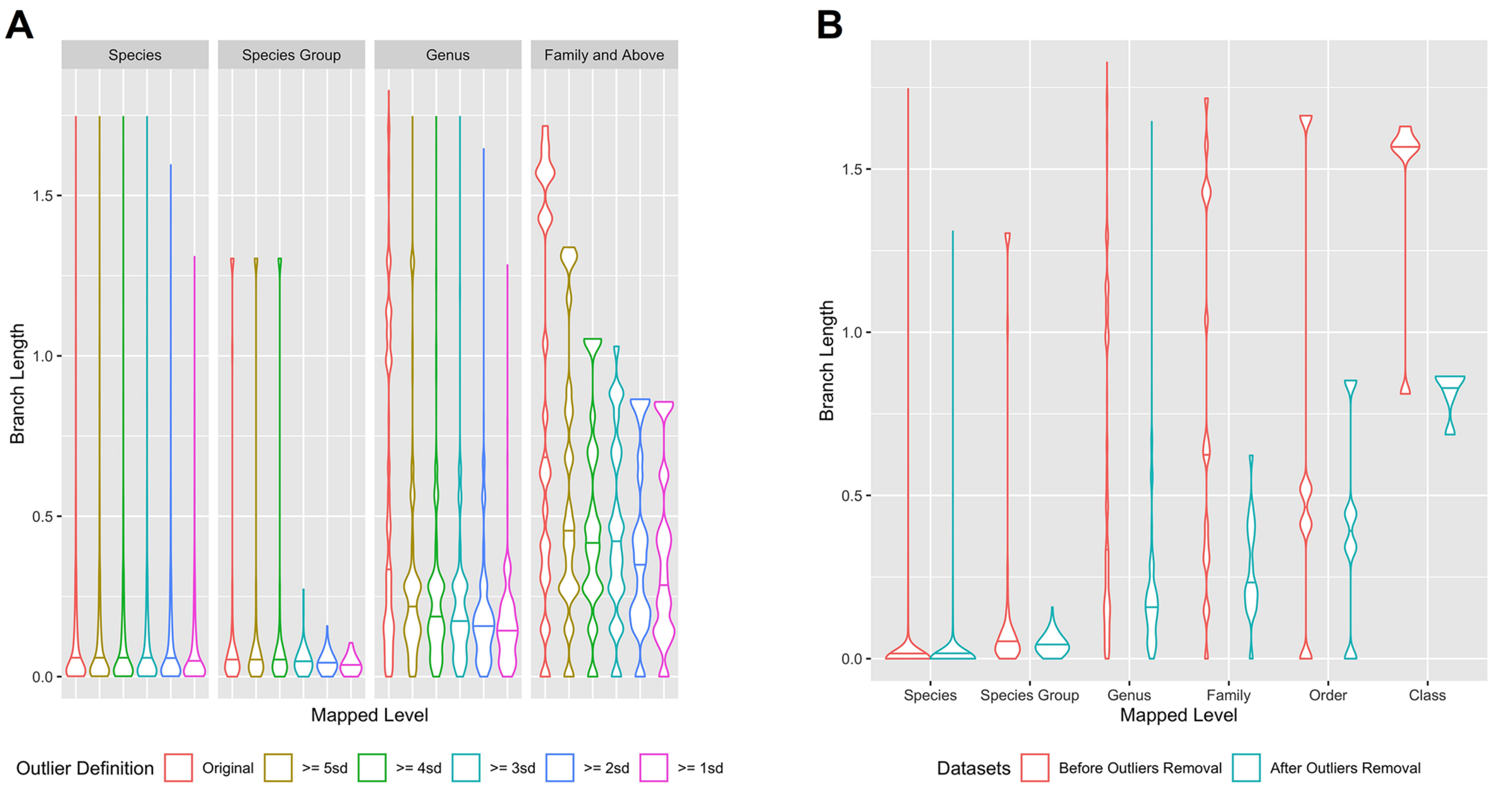

Fig. 2. (A) Changes in branch length distribution after removal of potential outlier tips defined using different thresholds. The figure is faceted by the taxonomic rank at which a query species could be mapped to the original GTDB phylogeny. For the first facet, species that were mapped with a branch length of 0 (i.e., inferred from only one reference tip) were excluded from the plot for better evaluation of the effects of removing outliers. (B) Overall comparison of branch length distribution after removal of all outliers. Final threshold used in this study are: ≥1sd for species level; ≥2sd for species group level; and ≥2sd for genus level and above.

Table 1. Summary of average number of reference tips and mean branch length for query species mapped at different ranks when different threshold values were applied.

Defined Potential Outliers	Species		Species Group		Genus		Family		Order		Class	
	n_refs	length	n_refs	length	n_refs	length	n_refs	length	n_refs	length	n_refs	length
Original	1.21	0.0083	18.69	0.1897	158.40	0.5432	126.88	0.7629	412.00	0.6486	3721.40	1.4296
≥ 5 sd	1.21	0.0083	18.68	0.1750	157.57	0.2911	126.00	0.4362	411.00	0.5273	3708.20	1.1142
≥ 4 sd	1.21	0.0082	18.68	0.1750	157.45	0.2322	125.69	0.3500	410.50	0.4898	3698.80	0.9375
≥ 3 sd	1.21	0.0082	18.37	0.0492	157.00	0.2087	125.19	0.3426	406.75	0.4733	3655.80	0.8354
≥ 2 sd	1.20	0.0076	17.60	0.0416	155.39	0.1878	119.88	0.2776	376.25	0.4093	3543.60	0.8184
≥ 1 sd	1.18	0.0060	16.29	0.0339	148.51	0.1530	115.00	0.2591	371.50	0.3983	3310.20	0.6747
Final[a]	1.18	0.0060	17.60	0.0416	155.39	0.1878	119.88	0.2776	376.25	0.4093	3543.60	0.8184

[a] Final threshold used in this study are: ≥1sd for species level; ≥2sd for species group level; and ≥2sd for genus level and above.

Reference

1. Parks, D.H., et al., *A standardized bacterial taxonomy based on genome phylogeny substantially revises the tree of life.* Nat Biotechnol, 2018. **36**(10): p. 996-1004.
2. Cock, P.J., et al., *Biopython: freely available Python tools for computational molecular biology and bioinformatics.* Bioinformatics, 2009. **25**(11): p. 1422-3.
3. Paradis, E., J. Claude, and K. Strimmer, *APE: Analyses of Phylogenetics and Evolution in R language.* Bioinformatics, 2004. **20**(2): p. 289-90.
4. Schliep, K.P., *phangorn: phylogenetic analysis in R.* Bioinformatics, 2011. **27**(4): p. 592-3.
